# The relationship between self-reported poor mental health and complete tooth loss among the US adult population in 2019

**DOI:** 10.3389/froh.2024.1363982

**Published:** 2024-03-28

**Authors:** Tasha Powell, Heather Taylor

**Affiliations:** ^1^ Comprehensive Care: Dental Hygiene, Indiana University School of Dentistry, Indianapolis, IN, United States; ^2^ Health Policy and Management, Richard M. Fairbanks School of Public Health, Indianapolis University, Indianapolis, IN, United States

**Keywords:** mental disorders, oral health, dentistry, complete tooth loss, MEPS

## Abstract

**Objective:**

Very little is known about the association between poor mental health and poor oral health outcomes in the United Sates. This study investigated the prevalence of complete tooth loss among those with and without perceived poor mental health in a nationally representative sample of noninstitutionalized U.S. adults.

**Methods:**

Using a cross-sectional study design, we analyzed the 2019 Medical Expenditures Panel Survey to determine the unweighted and weighted prevalence of complete tooth loss among adults. Chi-squared and multivariate logit regression with marginal effects were used to measure the association between complete tooth loss and perceived poor mental health, controlling for respondent characteristics.

**Results:**

The prevalence of adults (ages 18 and older) experiencing complete tooth loss was 6% (95% CI: 5.6–6.4). Individuals who have perceived poor mental health were 1.90 percentage points (pps) more likely to report missing all their natural teeth (*P *= 0.006: 95% CI: 0.5–3.3). Other relevant predictors of complete tooth loss included current smoking status (5.9 pps; 95% CI: 4.5 to 7.2) and secondary education (−6.4 pps (95% CI: −7.0 to −4.8).

**Conclusions:**

Overall, self-reported poor mental health was found to be associated with a greater likelihood of reporting complete tooth loss. Findings from this study underscore the need for greater integration of care delivery between behavioral health specialists and dental providers.

## 1 Introduction

Mental illnesses (i.e., conditions that affect cognition, emotion, and behavior) (1) and poor oral health (i.e., conditions and outcomes of oral diseases that include dental caries, periodontal (gum) disease, tooth loss, and oral cancer) (2) are two seemingly distinct domains, but emerging evidence suggests an intricate association (3). Existing studies suggest that individuals who experience a mental illness have a higher risk of poor oral health than those in the general population (4, 5). This may, in part, be due to certain risk factors among those with mental illness. For example, individuals who experience mental illness are frequently prescribed psychotropic medications, which have numerous negative oral side effects, such as xerostomia (dry mouth) and mouth ulcers (6, 7). Those who experience mental illness may also struggle to manage typical daily functions, such as remembering to stick to a schedule and maintaining their personal hygiene, including oral hygiene (8). Furthermore, they are also more likely than the general population to (1) consume diets high in fermentable carbohydrates which contributes to the initiation and progression of dental caries (3), (2) have comorbidities such as alcohol and substance use which is associated with dental caries, oral pain, and tooth infection (9), and (3) smoke cigarettes which can lead to periodontal disease and tooth loss (10, 11).

Given the lifestyle habits and risk factors among those with mental illnesses, understanding oral health outcomes among this population becomes very important. If poor oral health remains unaddressed, those with mental illness may experience worsening levels of self-esteem and reduced quality of life, further perpetuating complications related to their mental illness (3). However, most of our current understanding of the association between mental illnesses and oral health has been conducted in countries such as the United Kingdom, Australia, and Taiwan (3, 6, 7, 11–15). Of the existing U.S. based scientific literature, studies have limited their analyses to a small subset of a broad array of existing mental illness diagnoses, such as severe mental illness or depression (16–22). Nevertheless, there are many other mental illnesses, such as anxiety, which are related to bruxism (excessive teeth grinding) and attrition (tooth wear) and can also contribute to worse oral health (23, 24). Thus, there is a need to broadly understand whether, and to what extent, mental illnesses (including those less severe) are related to oral health so that population-level interventions can be targeted appropriately to address co-occurring health needs.

The purpose of this cross-sectional study is to determine the prevalence of complete tooth loss, a measure of poor oral health, among those experiencing mental illness in the United States. Specifically, this study will examine the relationship between self-reported poor mental health and complete tooth loss among a nationally representative survey of U.S. civilian noninstitutionalized adults (25). Complete tooth loss reflects a lifetime of dental disease and a history of (or absence) of treatment (26). We hypothesize that adults with poor mental health are more likely to experience complete tooth loss when compared to the general population. Findings from this study may inform policy on vulnerable populations and will also be of interest to behavioral health specialists, caregivers, dental professionals, and social workers who care for individuals with mental illness and wish to improve their oral and overall health (3).

## 2 Methods

Data for this cross-sectional study were derived from the 2019 Medical Expenditure Panel Survey (MEPS) data (27). We followed the STROBE guidelines for the reporting of this cross-sectional observational study (28). MEPS is a nationally representative annual survey of noninstitutionalized persons across the United States. This survey utilizes a subsample of participants from the previous year's National Health Interview Survey, conducted by the National Center for Health Statistics (29, 30). Thus, data collection for this analysis occurred between January and December of 2018 (30, 31). We used data from the MEPS household component (MEPS-HC), which collects participant information via interviews on demographic characteristics, health conditions (including dental services), insurance status, and quality of life data (29). Previous studies have reported on the validity of measures used in MEPS, including those used in the household component (32, 33).

In 2019, 28,512 persons were included in MEPS-HC (27). Because we were only interested in adults for our analysis, we excluded anyone who was under the age of 18 (*n* = 6,558). Since MEPS-HC data are publicly available and deidentified, this study was deemed *exempt* from IRB approval.

Our dependent variable, complete tooth loss, was constructed as a binary indicator based on whether or not an individual reported they “lost all upper and lower teeth” in the MEPS-HC interview. Respondents who reported “Yes” to having lost all upper and lower teeth were coded as “1” and labeled as having “complete tooth loss”. All individuals who reported “No” to having “lost all upper and lower teeth” were coded as “0”. Individuals who were “unable” or “refused” to answer this question were recoded as having a missing value for this variable (*n* = 517) but these observations remained in the sample.

Our main explanatory variable was whether an adult perceived their mental health to be poor. Respondents of the MEPS-HC interview were asked: “In general, would you say your mental health is excellent, very good, good, fair, or poor?” Respondents who reported “poor” or “fair” mental health were coded as “1”, while individuals who reported “good”, “very good”, and “excellent” mental health were coded as “0”. For individuals that were “unable” or “refused” to respond to this question, we recoded their responses to missing values (*n* = 388) but these observations remained in the sample.

Other independent factors served as controls in our analysis given their known relationship to healthcare utilization according to Andersen's Behavioral Model of Health Services Use (34). These control variables included gender (male, female), relationship status (married, widowed, divorced, separated, never married), income as percentage of the federal poverty line (FPL) [poor/negative (at FPL or below), near poor (FPL to less than 125% of the FPL), low income (125% to less than 200% FPL), middle income (200% to less than 400% FPL), high income (400% or greater FPL)], unemployed (yes, no), and health insurance coverage (private, public only, uninsured). We considered the following variables as confounding factors that are likely related to both our dependent and main explanatory variable: age (18–24; 25–34; 35–44; 45–54; 55–64; 65–74; 75+), race/ethnicity (Hispanic, non-Hispanic white, non-Hispanic black, non-Hispanic Asian, non-Hispanic other races or multiple races), education [no degree, high school/General Equivalency Diploma (GED)some college], private dental insurance (yes, no), general health status (excellent, very good, good, fair, poor,) current smoker status (yes, no), and whether they “cannot afford dental care” (yes, no). In most cases, we kept variables as they were defined, measured, and recorded within the MEPS datasets. For a few variables, data was reconstructed for the purposes of this analysis based on the distribution of responses per category and ease of interpretation (age, education, poor mental health).

### 2.1 Analysis

First, we tabulated unweighted and weighted descriptive statistics for each variable of interest included in our study, as well as the prevalence of complete tooth loss. Next, using Chi-Square tests, we examined the bivariate relationships between complete tooth loss and perceived mental health and respondent characteristics. To address potential sources of bias, we considered measures of collinearity and used a stepwise approach while building our adjusted regression model. The mean variable inflation factor (VIF) for included variables was 1.31 (A VIF above 5 is considered to indicate variables to be highly correlated and problematic to estimating unbiased regression coefficients). Because odds ratios cannot be compared across different population samples or interpreted as absolute effects, we chose to estimate marginal effects (35). Thus, we estimated the marginal effects of perceived mental health and each respondent characteristic included in the multivariate logit regression model. Marginal effects can be interpreted as the absolute risk difference of each independent variable when holding all other covariates at their mean value. Corresponding 95% confidence intervals were calculated for all regression estimates, and findings were considered statistically significant if *p*-values were below 0.05. For all analyses, MEPS survey weights were applied to obtain accurate weighted estimates and standard errors, using Stata 17 statistical software (36). MEPS survey weights adjust for survey nonresponse and disproportionate sampling of certain populations (37).

## 3 Results

A total of 21,954 non-institutionalized adults, representing 253,548,355 people, were included in this 2019 cross-sectional analysis. Descriptive statistics of the sample's characteristics are presented in Table 1. The prevalence of adults reporting poor mental health was 8.3% (95% CI: 7.8–8.8). The prevalence of complete tooth loss among all adults was 6.0% (95% CI: 5.6–6.4).

**Table 1:** Sample characteristics of U.S. adults, 2019 (*N* = 21,954).

Characteristic	Unweighted count	Total sample unweighted %	Total sample weighted % (95% CI^a^)
Reported poor mental health	1,972	9.1	8.3 (7.8–8.8)
Gender			
Female	11,667	53.1	51.6 (51.1–52.1)
Male	10,287	46.9	48.4 (47.9–49.0)
Ages			
18–24	2,237	10.2	11.6 (11.0–12.3)
25–34	3,405	15.5	17.7 (16.9–18.5)
35–44	3,467	15.8	16.2 (15.5–17.0)
45–54	3,441	15.7	16.2 (15.5–16.9)
55–64	3,828	17.4	16.7 (16.0–17.4)
65–74	3,213	14.6	12.6 (12.0–13.2)
75 and older	2,363	10.8	9.0 (8.5–9.5)
Race/Ethnicity			
Hispanic	4,458	20.3	16.4 (14.9–18.1)
Non-Hispanic White	12,530	57.1	62.5 (60.6–64.4)
Non-Hispanic Black	3,128	14.3	11.9 (10.8–13.0)
Non-Hispanic Asian	1,148	5.2	6.2 (5.3–7.2)
Non-Hispanic other race or multiple races	690	3.1	3.0 (2.7–3.4)
Relationship status			
Married	10,952	49.9	51.2 (50.1–52.3)
Widowed	1,635	7.5	6.3 (5.9–6.7)
Divorced	2,765	12.6	11.3 (10.7–11.9)
Separated	527	2.4	2.0 (1.8–2.2)
Never married	6,070	27.7	29.2 (28.3–30.2)
Education			
No degree	3,225	14.8	12.1 (11.3–12.9)
High school/GED^b^	10,054	46.2	44.6 (43.4–45.8)
Some college	8,499	39.0	43.3 (41.9–44.8)
Income^c^			
Poor/Negative	3,171	14.4	10.5 (9.8–11.3)
Near poor	994	4.5	3.7 (3.3–4.1)
Low income	3,059	13.9	12.4 (11.6–13.2)
Middle income	6,297	28.7	28.8 (27.6–30.0)
High income	8,433	38.4	44.6 (43.0–46.3)
Unemployed	8,665	40.4	35.3 (34.3–36.3)
Cannot afford dental care	2,691	12.5	11.3 (10.7–12.0)
Private dental insurance			
Yes	13,035	40.0	45.8 (44.5–47.2)
No	8,687	60.0	54.2 (52.8–55.5)
Health insurance coverage			
Any private	13,642	62.1	68.6 (67.2–69.9)
Public only	6,394	29.1	24.0 (23.0–25.1)
Uninsured	1,918	8.7	7.4 (6.8–8.1)
Complete tooth loss	1,558	7.3	6.0 (5.6–6.4)
General health status			
Excellent	3,183	18.4	20.2 (19.3–21.0)
Very good	6,135	35.5	37.7 (36.8–38.7)
Good	5,391	31.2	29.7 (28.8–30.6)
Fair	2,082	12.1	10.1 (9.6–10.7)
Poor	492	2.9	2.2 (2.0–2.5)
Current smoker			
Yes	3,112	14.5	13.8 (13.0–14.5)
No	18,326	85.5	86.2 (85.5–87.0)

Medical Expenditure Panel Survey (MEPS), 2019.

Weighted to population levels using sampling weights from the MEPS.

^a^ Confidence Interval calculated as *β* ± (1.96 × standard error).

^b^ GED, U.S. general equivalency diploma.

^c^ Income reported as a percentage of the Federal Poverty Line (FPL). Negative/poor (at FPL or below), near-poor income (between FPL and less than 125% of FPL), low income (indicates 125% FPL to less than 200% FPL), middle income (indicates 200% FPL to less than 400% FPL), and high income (at or above 400% of the FPL).

Table 2 displays the bivariate relationship between complete tooth loss and individual-level factors, including self-reported poor mental health. The relationship between poor mental health and complete tooth loss was found to be statistically significant (*β* = 0.131; *p*-value < 0.001). Statistically significant relationships were also found between complete tooth loss and income (*p* < 0.001), education (*p* < 0.001), private dental insurance (*p* < 0.001), and smoking status (*p* < 0.001).

**Table 2:** Bivariate relationship between complete tooth loss and individual level factors (*N* = 21,954).

Characteristic	Proportion	*P*-value	(95% CI^a^)
Reported poor mental health	0.131	<0.001	(0.115–0.149)
Gender		0.0892	
Female	0.063		(0.058–0.068)
Male	0.057		(0.051–0.063)
Age		<0.001	
18–24	0.015		(0.009–0.024)
25–34	0.013		(0.981–0.991)
35–44	0.021		(0.016–0.029)
45–54	0.039		(0.032–0.046)
55–64	0.070		(0.060–0.080)
65–74	0.132		(0.118–0.148)
75 and older	0.208		(0.185–0.232)
Race/ethnicity		<0.001	
Hispanic	0.044		(0.037–0.054)
Non-Hispanic White	0.066		(0.061–0.072)
Non-Hispanic Black	0.063		(0.053–0.075)
Non-Hispanic Asian	0.027		(0.018–0.040)
Non-Hispanic other race or multiple races	0.069		(0.049–0.096)
Income^b^		<0.001	
Poor/negative	0.110		(0.097–0.123)
Near poor	0.138		(0.111–0.171)
Low income	0.094		(0.081–0.109)
Middle income	0.063		(0.056–0.070)
High income	0.031		(0.027–0.036)
Education		<0.001	
No degree	0.129		(0.116–0.144)
High school/GED^c^	0.072		(0.066–0.078)
Some college	0.028		(0.024–0.032)
Health insurance coverage		<0.001	
Any private	0.037		(0.033–0.042)
Public only	0.135		(0.124–0.146)
Uninsured	0.034		(0.025–0.046)
Unemployed	0.118		(0.109–0.127)
Private dental insurance		<0.001	
Yes	0.026		(0.022–0.030)
No	0.911		(0.905–0917)
Cannot afford dental care	0.051	<0.001	(0.041–0.062)
General health status		<0.001	
Excellent	0.018		(0.014–0.023)
Very good	0.037		(0.032–0.043)
Good	0.079		(0.071–0.088)
Fair	0.141		(0.124–0.160)
Poor	0.196		(0.161–0.235)
Current smoker		<0.001	
Yes	0.120		(0.109–0.133)
No	0.950		(0.945–0.954)

Medical Expenditure Panel Survey (MEPS), 2019.

Weighted to population levels using sampling weights from the MEPS.

^a^ Confidence Interval calculated as *β* ± (1.96 × standard error).

^b^ Income reported as a percentage of the Federal Poverty Line (FPL). Negative/poor (at FPL or below), near-poor income (between FPL and less than 125% of FPL), low income (indicates 125% FPL to less than 200% FPL), middle income (indicates 200% FPL to less than 400% FPL), and high income (at or above 400% of the FPL).

^c^ GED, U.S. general equivalency diploma.

To examine the magnitude of effect of each independent variable and our main explanatory variable (perceived poor mental health) we present marginal effects from our multivariate regression model in Table 3. Adults who self-reported poor mental health were 1.9 percentage points (pps) (95% CI: 0.5–3.3) more likely to have complete tooth loss than those self-reporting good mental health. One of the largest predictors (by effect magnitude) of complete tooth loss was current smoking status [5.9 (pps); 95% CI: 4.5–7.2]. Another relevant predictor (by effect magnitude) was having a secondary education, which was negatively associated with complete tooth loss [−6.4 pps (95% CI: −7.0 to −4.8)] as compared to individuals with no degree/diploma. Adults reporting fair general health status (3.8 pps; 95% CI: 2.4–5.2) or poor general health status (4.4 pps; 95% CI: 2.4–6.4) were more likely to report complete tooth loss compared to those reporting excellent general health status. Finally, higher age was positively associated with complete tooth loss, as adults above age 45 were 2.6 pps (95% CI: 1.3–3.8) more likely to report complete tooth loss compared to adults below age 25.

**Table 3:** Marginal effect of factors associated with complete tooth loss among U.S. adults 2019 (*N* = 16,906).

Characteristics	Complete tooth loss	
	dy/dx^a^	95% CI^b^
Reported poor mental health	0.019	0.005 to 0.033
Gender		
Female	−0.004	−0.011 to 0.003
Male	Reference	Reference
Ages		
18–24	Reference	Reference
25–34	−0.004	−0.008 to 0.016
35–44	0.011	−0.002 to 0.024
45–54	0.026	0.013 to 0.038
55–64	0.048	0.034 to 0.062
65–74	0.091	0.074 to 0.109
75 and older	0.111	0.087 to 0.135
Race/ethnicity		
Hispanic	Reference	Reference
Non-Hispanic White	0.020	0.009 to 0.031
Non-Hispanic Black	0.015	0.003 to 0.029
Non-Hispanic Asian	−0.001	−0.023 to 0.057
Non-Hispanic other race or multiple races	0.032	0.006 to 0.057
Relationship status		
Married	Reference	Reference
Widowed	0.009	−0.002 to 0.020
Divorced	0.006	−0.004 to 0.016
Separated	<0.001	−0.021 to 0.021
Never married	−0.019	−0.030 to −0.008
Education		
No degree	Reference	Reference
High school/GED^c^	−0.039	−0.056 to −0.024
Some college	−0.064	−0.070 to −0.048
Unemployed	0.005	−0.006 to 0.017
Income^d^		
Poor/negative	Reference	Reference
Near poor	0.014	−0.009 to 0.037
Low income	−0.007	−0.024 to 0.009
Middle income	−0.010	−0.025 to 0.005
High income	−0.035	−0.049 to −0.020
Cannot afford dental care	−0.030	−0.038 to −0.021
Private dental insurance		
Yes	−0.013	−0.022 to −0.002
No	Reference	Reference
Health insurance coverage		
Any private	Reference	Reference
Public only	0.012	0.003 to 0.021
Uninsured	<0.001	−0.018 to 0.0184
General health status		
Excellent	Reference	Reference
Very good	0.012	0.0005 to 0.023
Good	0.027	0.0149 to 0.0398
Fair	0.038	0.024 to 0.052
Poor	0.044	0.024 to 0.064
Current smoker		
Yes	0.059	0.045 to 0.072
No	Reference	Reference

Medical Expenditure Panel Survey (MEPS), 2019.

Weighted to population levels using sampling weights from the MEPS.

^a^ dy/dx—Marginal effects are the absolute risk difference attributable for each characteristic, holding all other covariates at the mean.

^b^ Confidence Interval calculated as *β* ± (1.96 × standard error).

^c^ GED, general equivalency diploma.

^d^ Income reported as a percentage of the Federal Poverty Line (FPL). Negative/poor (at FPL or below), near-poor income (between FPL and less than 125% of FPL), low income (indicates 125% FPL to less than 200% FPL), middle income (indicates 200% FPL to less than 400% FPL), and high income (at or above 400% of the FPL).

## 4 Discussion

This study sought to determine the prevalence of complete tooth loss and examine its relationship with perceived poor mental health among adults within the United States. Overall, we found the prevalence of complete tooth loss among a nationally representative sample of adults was 6.0%. Furthermore, as hypothesized, we found that complete tooth loss was positively associated with self-reported poor mental health, even when controlling of other respondent characteristics.

Several studies have examined the prevalence of complete tooth loss and reported higher rates in the United States. One study assessed 2017 MEPS data and reported the prevalence of complete tooth loss to be 11.4% while another study assessed MEPS data over time (2015–2018) and measured the prevalence at 12.9% (38, 39). While these studies report a prevalence rate higher than what we found in this current study, these previous studies were restricted to a subset of the population, those over the age of 50. This current study examined the prevalence of complete tooth loss among all adults over the age of 18, hence why our prevalence rate is nearly half of what has been reported in past MEPS analyses.

In this current study, we found a significant bivariate relationship between those self-reporting poor mental health and complete tooth loss in the U.S. This relationship remained significant even after controlling for other factors in our multivariate regression models, as evident by the higher likelihood of complete tooth loss among those with perceived poor mental health. This finding is similar to studies which have examined the relationship between mental illness and tooth loss in other countries (3, 18). People with varying forms of mental illness often grapple with a myriad of challenges that can lead to poor oral outcomes such as complete tooth loss. These challenges may include side effects from commonly prescribed psychotropic drugs that often cause xerostomia (dry mouth), stigma and barriers to receiving dental care, a diet high in fermentable carbohydrates, and poor daily oral hygiene (40, 41).

One recent U.S. based survey study by Tiwari et al. (2022) similarly found that self-reported poor oral health was significantly related to self-reported poor mental health (2). Authors of this study reported that those with self-reported poor mental health were also statistically more likely to report unmet dental needs compared to their counterparts (2). Findings from this current study go a step further beyond unmet dental need and demonstrate that self-reported poor mental health is also positively related to the likelihood of experiencing measurable poor oral health outcomes (complete tooth loss). Tiwari et al. (2022) found that the majority of individuals who reported poor mental health did nothing about their oral health symptoms (2). These oral health symptoms, if not addressed appropriately, can lead to an increase in debilitating dental diseases, and eventual tooth loss (3, 40). For instance, one study revealed that over a third (−35%) of adults with mental illness require dental extractions due to delayed dental care brought on by unaddressed dental caries or infection (42). As more extractions are needed, individuals may eventually experience full edentulism (missing all natural teeth) and may subsequently need to consider dentures. Because dentures cannot perfectly stimulate true tooth function and form, these prostheses may perpetuate poor mental health by exacerbating low self-esteem and social withdrawal (3). Thus, the relationship between poor mental health and poor oral health is likely cyclical and pervasive in nature.

Beyond self-reported poor mental health, we also found smoking status to be the largest positive predictor (in magnitude) of complete tooth loss. Nearly forty percent of non-institutionalized persons with mental illnesses smoke (43). Furthermore, the smoking rate is twice as high among individuals with depression than the general population (44, 45). As mentioned, individuals who struggle with mental illness often have poor oral hygiene which can be exacerbated by social risks factors such as smoking. Smoking has a significant negative effect on oral health and can lead to periodontal disease, infection, and higher rates of dental extractions (3, 42, 46). Given the strong correlation between smoking and oral and mental health, medical and dental providers should seek ways to coordinate care for mutual patients who suffer from mental illnesses and engage in risky behaviors, such as smoking. By better addressing these habits and providing tobacco cessation help, providers may be able to make improvements in individual- and population-level mental and oral health.

Finally, some evidence suggests that those with mental illness face significant barriers to accessing dental care. For instance, some healthcare providers stigmatize patients who have mental illnesses, leading to excessively delayed treatment, if not denying care due to unconscious bias or inadequate behavioral health training that would allow providers to effectively manage patients and provide optimal care (47). Also, persons with mental illness often have an altered cognitive state and present with poor communication skills creating other barriers to dental care (16). These barriers may exacerbate or worsen their individual oral health among those with mental illness (48). Future research should continue to analyze these relationships, particularly within vulnerable communities who experience significant health disparities. It is important for policymakers, behavioral health specialists, caregivers, dental professionals, and social workers who manage or care for individuals with mental illness to understand whether, and to what extent, mental illness is related to oral health so that interventions can be targeted appropriately and needs can be better addressed.

While our study was the first to examine the association between perceived poor mental health and complete tooth loss in the U.S., there are several study limitations to note. First, our cross-sectional study design inhibits internal validity and therefore the ability to determine causality (49). Second, we used self-reported survey data which lends itself to recall and social desirability biases. Third, “mental health” is a broad term that includes many factors as well as the absence of a broad arrange of conditions and disorders (50). Nevertheless, research suggests that self-reported mental health measures are good proxy variables for clinically definitions of mental health (51). Fourth, given MEPS sampling design of non-institutionalized individuals, we know very little about the prevalence or extent of complete tooth loss among institutionalized adults, who are statistically more likely to have mental illnesses (52, 53). Thus, while our data are nationally representative, our findings cannot be generalized to the institutionalized U.S. adult population. Given this limitation, we likely underestimated the association between perceived poor mental health and complete tooth loss (52, 53). Fifth, our findings may also be explained by reverse causality, this is, that complete tooth loss drives perceived poor mental health. Additional prospective research that studies these relationships overtime is needed to tease out the true mechanisms of action within this observed relationship. And finally, survey data can be subject to nonresponse bias. However, we applied appropriate MEPS sampling weights to account for this potential bias in nonresponse.

## 5 Conclusion

In summary, our findings address relevant gaps in the literature related to mental illness and complete tooth loss among the U.S. adult population. The observed positive association between poor mental health and complete tooth loss underscores the importance of further exploring the interplay between mental illness and oral health.

## Data Availability

Publicly available datasets were analyzed in this study. This data can be found here: MEPS HC-192 2016 Full Year Consolidated Data File. https://meps.ahrq.gov/data_stats/download_data/pufs/h192/h192doc.shtml.
